# Supplementary material for: Red Sea Bream Iridovirus (RSIV) Kinetics in Rock Bream (Oplegnathus fasciatus) at Various Fish-Rearing Seawater Temperatures
Source: Animals (Basel). 2022 Aug 4;12(15):1978. doi: 10.3390/ani12151978 (PMC9367270; doi:10.3390/ani12151978)
Supplement: Supplementary file 1 [file animals-12-01978-s001.zip › animals-1810286-supplementary.pdf]

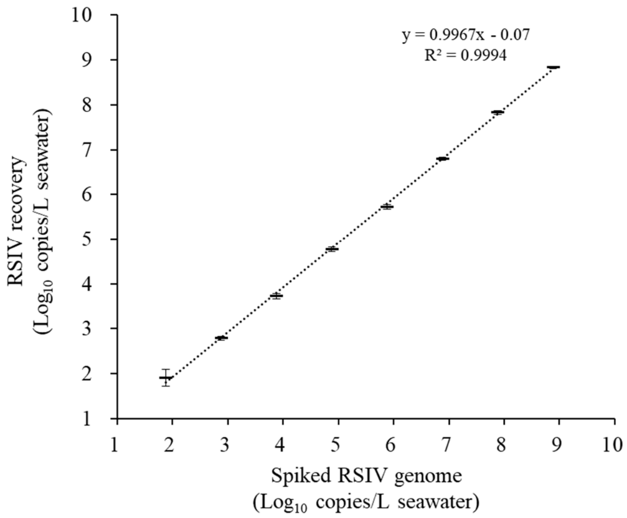

Figure S1. Relationship between the spiked number and recovery number of red sea bream iridovirus (RSIV) genome as determined by iron-based flocculation and TaqMan probe-based real-time PCR. The error bars represent the standard deviation of the mean (n = 3).

Table S1. Red sea bream iridovirus (RSIV) genome recovery efficiency from RSIV-spiked artificial seawater.

Initial copy number (Viral copies/L seawater)	Recovered concentration (Viral copies/L seawater)	Average
7.60E+08	6.27E+08<br>6.85E+08<br>6.86E+08	6.66E+08
7.60E+07	5.96E+07<br>6.62E+07<br>7.23E+07	6.60E+07
7.60E+06	6.31E+06<br>5.66E+06<br>6.71E+06	6.23E+06
7.60E+05	4.82E+05<br>5.95E+05<br>5.11E+05	5.29E+05
7.60E+04	5.99E+04<br>6.55E+04<br>5.13E+04	5.89E+04
7.60E+03	4.59E+03<br>5.95E+03<br>5.52E+03	5.35E+03
7.60E+02	6.25E+02<br>6.62E+02<br>5.51E+02	6.13E+02
7.60E+01	1.29E+02<br>5.70E+01<br>7.20E+01	8.60E+01
7.60E+00	Not detected<br>Not detected<br>Not detected	Not applicable
